# A high fat diet fosters elevated bisretinoids

**DOI:** 10.1016/j.jbc.2023.104784

**Published:** 2023-05-04

**Authors:** Hye Jin Kim, Jin Zhao, Jose L. Walewski, Janet R. Sparrow

**Affiliations:** 1 Departments of Ophthalmology, Columbia University Medical Center, New York New York, USA; 2 Departments of Medicine, Columbia University Medical Center, New York New York, USA; 3 Departments of Pathology and Cell Biology, Columbia University Medical Center, New York New York, USA

**Keywords:** diet-induced obesity, vitamin A, retina, phosphatidylethanolamine, retinol-binding protein 4

## Abstract

High dietary fat intake is associated with metabolic dysregulation, but little is known regarding the effects of a high fat diet (HFD) on photoreceptor cell functioning. We explored the intersection of an HFD and the visual cycle adducts that form in photoreceptor cells by nonenzymatic reactions. In black C57BL/6J mice and albino C57BL/6J^c2j^ mice raised on an HFD until age 3, 6, or 12 months, chromatographically quantified bisretinoids were increased relative to mice on a standard diet. *In vivo* measurement of fundus autofluorescence, the source of which is bisretinoid, also revealed a significant increase in the HFD mice. Additionally, mice provided with a diet high in fat presented with elevated retinol-binding protein 4, the protein responsible for transporting retinol in plasma. Vitamin A was elevated in plasma although not in ocular tissue. Bisretinoids form in photoreceptor cell outer segments by random reactions of retinaldehyde with phosphatidylethanolamine. We found that the latter phospholipid was significantly increased in mice fed an HFD *versus* mice on a control diet. In leptin-deficient *ob/ob* mice, a genetic model of obesity, plasma levels of retinol-binding protein 4 were higher but bisretinoids in retina were not elevated. Photoreceptor cell viability measured as outer nuclear layer thickness was reduced in the *ob/ob* mice relative to WT. The accelerated formation of bisretinoid we observed in diet-induced obese mice is related to the high fat intake and to increased delivery of vitamin A to the visual cycle.

Throughout the lifetime of an individual, vitamin A aldehyde adducts (bisretinoids) form randomly and nonenzymatically by the reaction of retinaldehyde with photoreceptor outer segment lipid—specifically, phosphatidylethanolamine (PE) (1). Bisretinoids are transferred to retinal pigment epithelial (RPE) cells within phagocytosed outer segment disks and accumulate as lipofuscin (2, 3, 4, 5, 6). These bisretinoid fluorophores can be measured chromatographically (7, 8). In addition, since they are the source of short wavelength fundus autofluorescence (SW-AF; 488 nm, blue excitation) that is detected spectrophotometrically (9) and imaged noninvasively by confocal fluorescence scanning laser ophthalmoscopy (10), methods for quantifying fundus autofluorescence (quantitative fundus autofluorescence; qAF) in human subjects/patients and mice are also in use (11, 12, 13, 14, 15, 16).

Bisretinoids exhibit properties having adverse consequences for cells. For instance, these compounds are photosensitizers having a propensity to generate reactive forms of oxygen including superoxide anion and singlet oxygen (17, 18). These reactive species subsequently add to carbon-carbon double bonds within the side-arms of the bisretinoid molecule; the latter eventually photodegrade, releasing damaging aldehyde- and dicarbonyl-bearing molecular fragments. Although all healthy eyes accumulate bisretinoids with age (14, 15, 19, 20, 21, 22), there are some variables that are known to influence the quantities accrued. For instance, levels of bisretinoid at any given time reflect the balance between formation in photoreceptor cells *versus* photodegradative loss (15, 23). It is also known that the rate of bisretinoid formation can be modulated by visual cycle kinetics. Thus, limiting delivery of vitamin A to RPE (24, 25) or reducing the activity of the isomerase RPE65 by a murine gene variant (26), null mutation (27, 28), or by small molecules that target RPE65 (29, 30, 31) also reduces or abrogates bisretinoid formation. Similar effects are observed in humans carrying deficiencies in other proteins of the visual cycle including lecithin retinol acyltransferase, cellular retinaldehyde-binding protein, and 11-*cis* retinol dehydrogenase (32, 33). Conversely, disease-causing mutations in ATP-binding cassette transporter A4 and retinol dehydrogenases cause mishandling of vitamin A aldehyde and accelerated formation of bisretinoid (34, 35, 36, 37).

Noninvasive measurement of bisretinoid by qAF has also revealed higher levels in individuals self reporting as female and White and lower in Asians and Blacks (14). Another variable is smoking; qAF is higher in subjects that identify as smokers (14). Nevertheless, the considerable difference in fundus autofluorescence intensities (∼3-fold range) (14) among individuals of similar age are not fully accounted for. Since supplementation with vitamin A in mice is known to increase the accumulation of bisretinoid lipofuscin (38) and a high fat diet (HFD) can increase serum vitamin A (39, 40), we have undertaken to determine whether dysregulation of vitamin A aldehyde in association with an HFD can lead to increased formation of the vitamin A aldehyde adducts (retinaldehyde-adducts, bisretinoids) that constitute the lipofuscin of retina and have adverse consequences for RPE and photoreceptor cells. We have studied two mouse models of obesity, obesity due to an HFD and obesity due to deficiency in leptin (*Lep*^*ob/ob*^; *ob/ob*), a hormone that is produced in adipose tissue and is involved in regulating energy expenditure and food intake (41).

## Results

### Bisretinoids in HFD-fed mice

**Figure 1:**
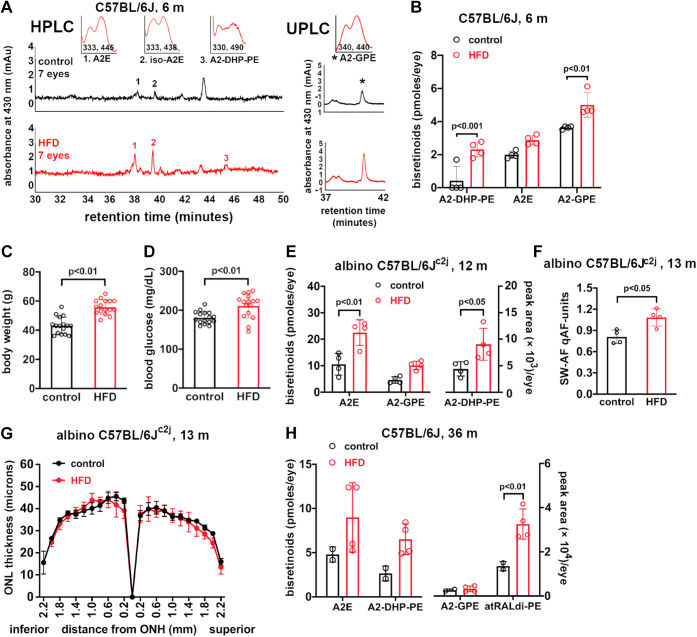
**Quantitation of bisretinoids in the eyes of diet-induced obese (high fat diet) mice and mice fed a standard diet (control).** Male black C57BL/6J and male albino C57BL/6Jc2j mice with ages as indicated in months (m). Bisretinoids A2E, iso-A2E, and A2-DHP-PE (A2-dihydropyridine-phosphatidylethanolamine) were quantified by HPLC. A2-GPE (A2-glycerophosphoethanolamine) was quantified using UPLC chromatograms. *A*, representative reverse phase HPLC chromatograms illustrating the detection of A2E, iso-A2E, and A2-DHP-PE (*left*). A2-GPE was detected by UPLC (*right*); age 6 months. Absorbance monitored at 430 nm; retention time in minutes. (Insets) UV/visible absorbance spectra corresponding to indicated peaks. *B*, chromatographic quantitation of bisretinoids. The value for A2E represents the sum of all-*trans* and *cis*-isomers; age 6 months. Individual values and means ± SD are plotted, n = 4. Each value is based on pooling of 1 to 7 eyes/sample. *C* and *D*, body weights (g) (*C*) and blood glucose (mg/dl) (*D*) in diet-induced obese (high fat diet, HFD) mice and mice fed a standard diet (control). Male albino C57BL/6J^c2j^ mice, age 12 months. Values are mean ± SD, n = 16. *p* values were determined by unpaired two-tailed *t* test. *E*, chromatographic quantitation of bisretinoids A2E, A2-GPE, and A2-DHP-PE at age 12 months. Individual values and means ± SD are plotted, n = 4. Each plotted value represents pooling of four eyes. *F*, quantitation of SW-AF (short-wavelength fundus autofluorescence; 488 nm excitation) in HFD-fed and standard diet mice; age 13 months. Individual values and means ± SD are plotted, n = 4. *G*, photoreceptor cell viability measured as outer nuclear layer (ONL) thickness in albino C57BL/6J^c2j^ mice fed the HFD from age 6 weeks to 13 months. ONL thicknesses (mean ± SEM) are plotted as distance from the optic nerve head (ONH), n = 8 eyes. *H*, chromatographic quantitation of bisretinoids (A2E, A2-DHP-PE, A2-GPE, and atRALdi-PE (all *trans* retinal dimer-phosphatidylethanolamine)); age 36 months (m). Two to ten eyes/each plotted value presented as pmoles/eye. Individual values and means ± SD are plotted; n = 2 to 4. Values for A2E are the sum of all-*trans*-A2E and *cis*-isomers of A2E (*B*, *E*, and *H*). *p* values determined by two-way ANOVA and Sidak’s multiple comparison test (*B*, *E*, and *H*) or unpaired two-tailed *t* test (*C*, *D*, and *F*). Extraction methods A (B, H) and B (E) were used. PE, phosphatidylethanolamine; UPLC, ultra-performance liquid chromatography.

To undertake studies of a relationship between obesity and bisretinoid levels, mice were raised on an HFD (60% kcal from fat) and comparison was made to mice raised on a standard control (10% kcal from fat) diet. Accordingly, we studied black C57BL/6J mice receiving an HFD or control diet beginning at 6 weeks of age with tissue collection at 6 months of age. The body weight of the HFD-fed mice was 46.4 g (±2.9, SD), a 37% increase over the mice on a standard diet (33.9 ± 2.7 g (mean ± SD)) (*p* < 0.001, unpaired two-tailed *t* test). Peaks in the HPLC and ultra-performance liquid chromatography (UPLC) chromatograms were identified as A2E, isoA2E, A2-DHP-PE, and A2GPE on the basis of UV-visible absorbance spectra and retention times (*R*_t_) that were consistent with authentic synthesized standards (7, 20, 42, 43). Accordingly, by reverse phase HPLC, we quantified A2E (the sum of all-*trans* and *cis*-isomers) and A2-DHP-PE by HPLC and A2-GPE by UPLC (Fig. 1*A*). In the eyes of HFD-fed mice, there was a 1.4-fold difference in A2-GPE (*p* < 0.01) and a 5.5-fold change in A2-DHP-PE (*p* < 0.001; two-way ANOVA, Sidak’s multiple comparisons test) (Fig. 1*B*). An independent *t* test conducted to compare total measured bisretinoid in the HFD-fed mice (10.2 ± 1.4) to total measured bisretinoid in the control mice (6.1 ± 0.9) revealed a statistically significant difference (*p* < 0.01) with a large effect size (d = 5.1).

Given that bisretinoids can undergo loss by photodegradation, we also compared albino C57BL/6J^c2j^ mice that were fed an HFD from age 6 weeks to 12 months of age with C57BL/6J^c2j^ mice on a standard diet. The final weight of the HFD-fed mice (55.9 ± 5 gm) was 30% greater than the weight of the control mice (43.2 ± 5.9 gm; mean ± SD) (*p* < 0.01, unpaired two-tailed *t* test) (Fig. 1*C*). Blood glucose was also elevated in these mice (HFD: 211 ± 31; control: 181 ± 15.9, mean ± SD, mg/dl) (*p* < 0.01, unpaired two-tailed *t* test) (Fig. 1*D*). The levels of A2E in obese C57BL/6J^c2j^ HFD-fed mice were more than 2-fold greater than in the mice fed the standard diet (HFD: 22.5 ± 4.8; control: 10.6 ± 4.1, mean ± SD; *p* < 0.01, two-way ANOVA and Sidak’s multiple comparisons test), and similar increases in the bisretinoid A2-DHP-PE (2-fold) were also measured (A2-GPE, HFD: 10.1 ± 1.6; control: 4.7 ± 1.2, mean ± SD; *p* > 0.05, Sidak’s multiple comparisons test) (A2-DHP-PE *p* < 0.05, Sidak’s multiple comparisons test) (Fig. 1*E*).

An independent *t* test reported a statistically significant difference (*p* < 0.01) between total measured bisretinoid in the HFD-fed mice (32.7 ± 6.3) *versus* control mice (15.3 ± 5.3). In this analysis, the effect size was large (d = 4.3). It was also clear that the increase in bisretinoid formation in the HFD-fed albino mice was not off-set by photodegradation in mice exposed to higher levels of intraocular light.

Fundus imaging to measure SW-AF noninvasively (qAF) (15), the source of which is bisretinoid, revealed that qAF was 35% higher in the HFD-fed mice (1.1 ± 0.12, mean ± SD) than in the control mice (0.8 ± 1.0, mean ± SD; *p* < 0.05, unpaired *t* test) (Fig. 1*F*). The effect size (d = 2.5) met the benchmark of a large effect. This increase in bisretinoid in the C57BL/6J^c2j^ mice was not associated with a change in photoreceptor cell viability. Specifically, no difference in outer nuclear layer (ONL) area was observed when mice receiving the HFD were compared to those receiving the standard diet at age 13 months (*p* > 0.05, unpaired two-tailed *t* test) (Fig. 1*G*).

In a study of C57BL/6J mice as old as 32 months, it has been demonstrated that scotopic and photopic a- and b-wave ERG amplitudes are reduced and ONL thicknesses are diminished (44). We were similarly interested how aging may affect the impact of an HFD. Thus, we also studied bisretinoids in extremely old (36 months) C57BL/6J mice. As shown in Figure 1*H*, in these 3-year-old mice reared on an HFD, atRALdi-PE was increased 2.4-fold in the diet-induced obese mice compared to mice on the standard (10% fat) diet (*p* < 0.01, two-way ANOVA, Sidak’s multiple comparisons test). Similarly, A2E (the sum of all A2E isomers) was 1.9-fold higher in the diet-induced obese mice relative to mice on the standard (10% fat) diet, while the increase in A2-DHP-PE was 2.5 greater (not statistically significant, *p* > 0.05). The mice receiving the HFD for 3 years weighed 56.6 ± 12.2 g as compared to a weight of 34.3 ± 2.3 g (mean ± SD) exhibited by mice on the control diet, but the weights of the HFD-fed 3-year-old and 1-year-old mice were similar. An expected age-related increase in bisretinoid was observed: total bisretinoid in the HFD-fed and control 3-year-old mice (the sum of A2E and A2-DHP-PE; 15.5 and 7.4 pmoles, respectively; Fig. 1*H*) was greater than in the 6-month-old black HFD-mice (the sum of A2E and A2-DHP-PE; 10.2 and 6.1 pmoles/eye, respectively; Fig. 1*B*).

### Plasma Rbp4

**Figure 2:**
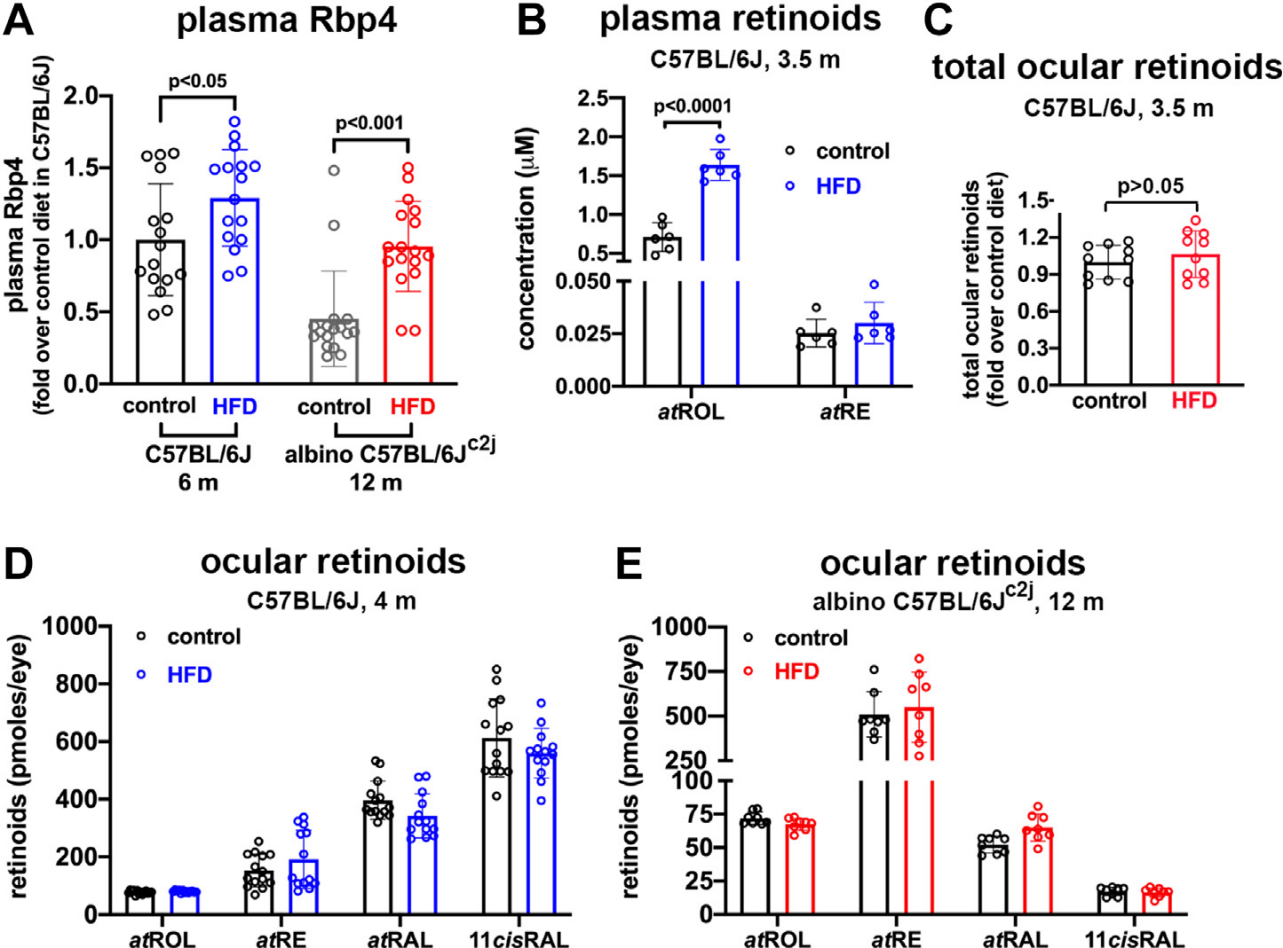
**Retinol binding protein 4 and retinoids measured in the plasma and eyes of male mice fed a high fat diet.** Controls were fed a standard diet. *A* and *B*, Rbp4 levels in C57BL/6J black mice fed an HFD from age 6 weeks to 6 months (m) and C57BL/6J^c2j^ albino mice fed an HFD from age 6 weeks to 12 months. Controls were fed a standard diet. Measurements were acquired by ELISA. Data are normalized as fold change in Rbp4 protein, HFD relative to standard diet level. Each sample value was calculated from duplicates. Individual values are plotted together with mean ± SD; n = 8. *B*, UPLC quantitation of plasma retinoids (all-*trans*-retinol *at*ROL; all-*trans*-retinyl palmitate, *at*RE) in C57BL/6J mice fed an HFD for 3.5 months. Values per single eyes are plotted together with mean ± SD; n = 6. *C*, retinoid levels in the eyes of dark-adapted male black C57BL/6J mice fed an HFD for 3.5 months. Total retinoids (all-*trans*-retinol, all-*trans*-retinyl ester, all-*trans*-retinal, and 11-*cis*-retinal) were summed. Data are normalized as fold change in total ocular retinoids, HFD relative to standard diet (control). Values per single eye are plotted together with mean ± SD, n = 10. *p* values were determined by unpaired two-tailed *t* test. *D*, retinoid levels in the eyes of light-adapted black C57BL/6J mice fed an HFD for 4 months. Retinoids *at*ROL, *at*RE, all-*trans*-retinal (*at*RAL), 11-*cis*-retinal (11*cis*RAL) are presented as picomoles per eye. Values per single eye are plotted together with mean ± SD; n = 6 to 14. *E*, retinoid levels in the eyes of albino C57BL/6J^c2j^ mice fed an HFD from age 6 weeks to 12 months. Retinoid extraction: method 1 (*D* and *E*). Values per single eye are plotted together with mean ± SD; n = 8. *p* values determined by two-way ANOVA and Sidak’s multiple comparison test (*A*, *B*, *D*, and *E*). HFD, high fat diet; UPLC, ultra-performance liquid chromatography.

For transport to cells, including the RPE, vitamin A forms a holo-enzyme complex with retinol-binding protein 4 (RBP4) and transthyretin (TTR) (45, 46). Thus, we measured Rbp4 levels in mouse plasma by ELISA using antibody to mouse Rbp4 protein. Rbp4 levels in the plasma of the HFD-fed C57BL/6J^c2j^ mice were elevated relative to control mice on a standard diet (*p* < 0.001, two-way ANOVA, Sidak’s multiple comparison test) at 12 months of age (Fig. 2*A*). We also measured Rbp4 levels in black C57BL/6J mice receiving an HFD or control diet beginning at 6 weeks of age with plasma collection at 6 months of age. In the plasma of HFD-fed mice, there was a 1.3-fold increase in Rbp4 (*p* < 0.05; 1-way ANOVA, Sidak’s multiple comparisons test) (Fig. 2*A*). The effect size (d = 4.5) was large.

### Retinoid levels in HFD-fed mice

To determine whether an HFD diet increases delivery of vitamin A to retina as a prelude to elevated bisretinoid lipofuscin formation, we also measured retinoids in plasma. In C57BL/6J mice receiving the HFD (age 14 weeks), there was a 2.2-fold increase (*p* < 0.0001; two-way ANOVA and Sidak’s multiple comparisons test) in all-*trans*-retinol (vitamin A) in plasma collected at euthanasia (Fig. 2*B*). The analysis also revealed a large effect size (d = 8.4). All-*trans*-retinyl ester was also detected in the plasma, but statistically significant differences were not observed (47).

In eyes harvested from light-adapted C57BL/6J mice fed an HFD *versus* standard diet (age 16 weeks), there were no statistically significant differences in the levels of retinoids including all-*trans*-retinol, all-*trans*-retinyl ester, all-*trans*-retinal, or 11-*cis*-retinal (Fig. 2*C*). Similarly, there were no statistically significant differences in the retinoid content of eyes acquired from HFD-fed albino C57BL/6J^c2j^ mice (light adapted; age 12 months) *versus* the control diet. In keeping with higher intraocular light levels in albino eyes, 11-*cis* retinal was less abundant in the albino (Fig. 2*D*). There was also no difference in total retinoid levels in dark-adapted black C57BL/6J mice fed an HFD (age 3.5 months) (Fig. 2*C*).

### PE assay

**Figure 3:**
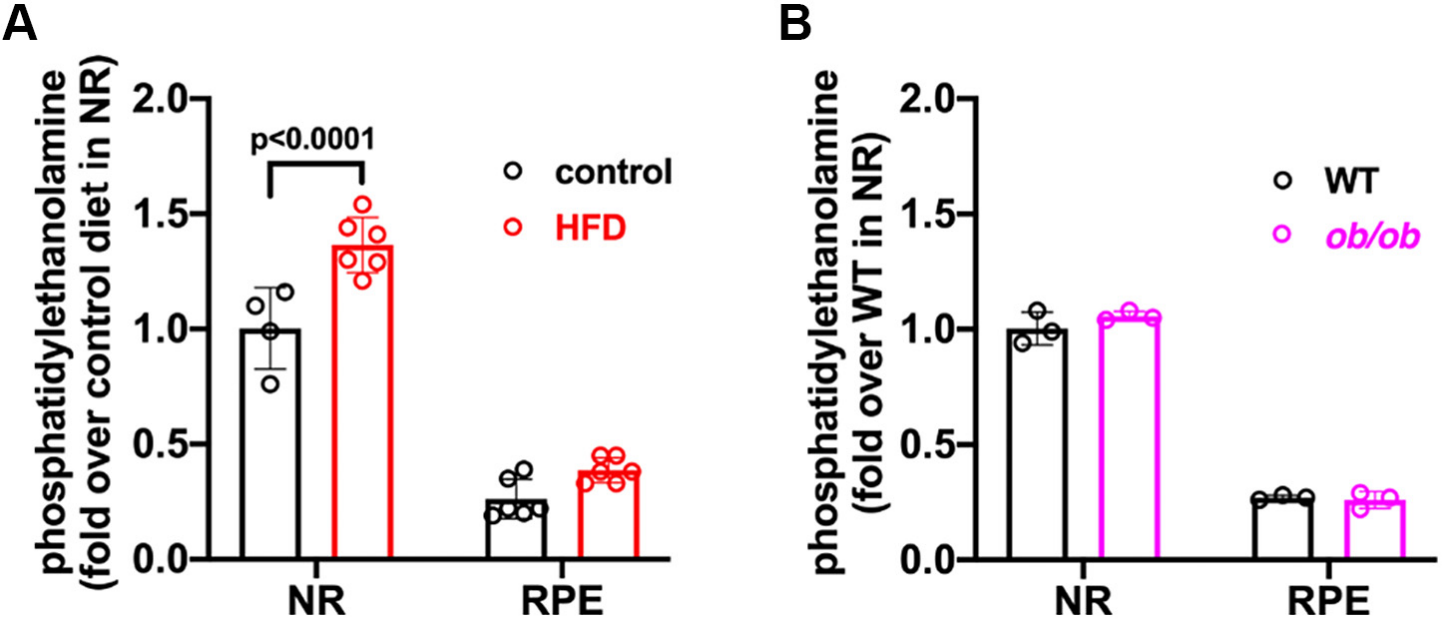
**Phosphatidylethanolamine measurements.** PE was assayed in neural retina (NR) and retinal pigment epithelium (RPE)/choroid of black C57BL/6J mice fed a high fat diet (HFD) from age 6 weeks to 4 months (*A*) and in *ob/ob* mice at age 3 months (*B*). Controls were fed a standard diet. Measurements were acquired by fluorescence assay. Values are plotted together with mean ± SD; n = 3 to 6. Means were not significantly different. *p* values determined by two-way ANOVA and Sidak’s multiple comparison test. PE, phosphatidylethanolamine.

PE is a neutral phospholipid consisting of a phosphatidyl group with an ester linked to ethanolamine. Since bisretinoids form in photoreceptor cell outer segments by random reactions of retinaldehyde with PE, we also assayed PE content. Since approximately 80% of the cells in mouse retina are photoreceptors (48, 49, 50) and in photoreceptor outer segments, PE constitutes 37.6 mol% of the phospholipids (51) we employed isolated neural retina for these measurements. We found that in black C57BL/6J mice fed an HFD, PE was 37% higher than in mice on the control diet (*p* < 0. 001; two-way ANOVA and Sidak’s multiple comparisons test) (Fig. 3*A*). In this analysis, the effect size was large (d = 3.6).

### Findings in ob/ob mice

Leptin-deficient *ob/ob* mice are a genetic model of obesity. *Ob/ob* mice become morbidly obese on a standard diet due to excessive food intake; the development of obesity is more rapid and severe than with HFD (52). Thus, we set out to determine whether this genetic model of obesity (*ob/ob*) on a C57BL/6J background is associated with elevated ocular retinoids and whether formation of vitamin A aldehyde adducts (bisretinoid lipofuscin) is increased.

**Figure 4:**
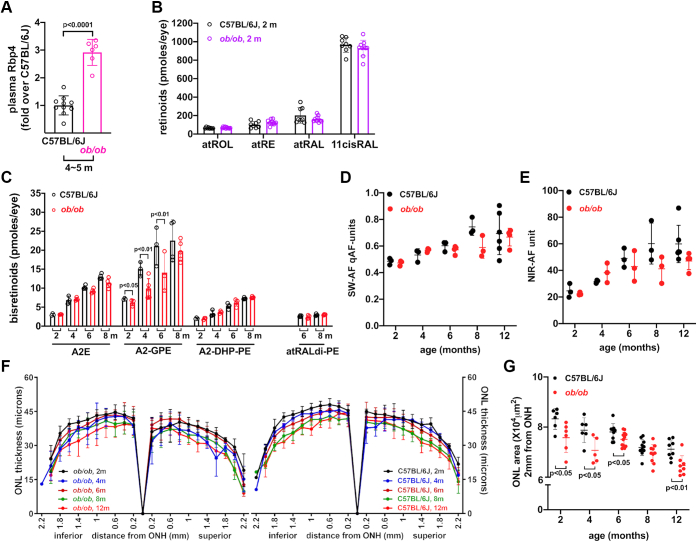
**Leptin-deficient black *ob/ob* mice and WT black C57BL/6J mice.** Mouse cohorts were gender matched (female/male). *A*, retinol binding protein 4 (Rbp4) in the plasma of *ob/ob* mice aged 5 months and C57BL/6J mice aged 4 months. Measurements were carried out by ELISA. Data are normalized as fold change in Rbp4 in *ob/ob* mice relative to C57BL/6J, n = 6 to 10. *p* value was determined by unpaired two-tailed *t* test. *B*, retinoid levels in the eyes of light adapted 2 months-old *ob/ob* and C57BL/6J mice. *at*ROL, all-*trans*-retinol; *at*RE, all-*trans*-retinyl ester; all-*trans*-retinal (*at*RAL), 11-*cis*-retinal (11*cis*RAL). Retinoid extraction was performed using method 2. Values were obtained from single eyes and are plotted together with mean ± SD, n = 8 to 10. *p* values were determined by two-way ANOVA and Sidak’s multiple comparison test. *C*, quantitation of bisretinoids in *ob/ob* and C57BL/6J mice at ages 2, 4, 6, and 8 months. Individual values and mean ± SD are plotted, n = 2 to 5 replicates. Extraction method B was used. *P* values determined by two-way ANOVA and Sidak’s multiple comparison test. *D* and *E*, quantitation of SW-AF (short-wavelength fundus autofluorescence; 488 nm excitation) and NIR-AF (near-infrared fundus AF; 790 nm excitation) at age 2, 4, 6, and 8 months. Individual values and mean ± SD are plotted, n = 3 mice. Means were not significantly different (one-way ANOVA and Tukey’s multiple comparison test). *F*, outer nuclear layer (ONL) thickness in *ob/ob* mice and C57BL/6J mice at ages 2, 4, 6, 8, and 12 months. ONL thickness is plotted as distance from optic nerve head (ONH), n = 6 to 12 eyes. *G*, ONL area calculated from ONL thickness measurements as described in Experimental procedures. Values are mean ± SD. *p* values were determined by one-way ANOVA and Tukey’s multiple comparison test. n = 6 to 12 eyes.

Within our cohort of black *ob/ob* mice, we observed the mice to be 42.0 ± 3.4 g at 2 months and as much as 71.1 ± 6.2 g at 8 months (mean ± SD). Plasma levels of Rbp4 protein were also higher in the *ob/ob* mice (Fig. 4*A*), as has been previously reported (52, 53, 54). In the *ob/ob* mice, Rbp4 was 2.9 higher than in WT C57BL/6J mice with a large effect size (d = 8.7) (*p* < 0.0001, unpaired two-tailed *t* test) (Fig. 4*A*).

In eyes collected from light-adapted *ob/ob versus* WT mice (age 2 months), there were no statistically significant differences in the levels of retinoids including all-*trans*-retinol, all-*trans*-retinyl ester, all-*trans*-retinal, or 11-*cis*-retinal (Fig. 4*B*). We measured retinal bisretinoids by HPLC. In a comparison of *ob/ob* and WT mice, no differences were observed in A2E (the sum of all isomers) nor in A2-DHP-PE. At 6 and 8 months of age, all-*trans*-retinal dimer-PE levels were sufficient for quantitation, but no differences were observed between *ob/ob* and C57BL/6J mice. Conversely, we observed consistently lower levels of A2-GPE in the *ob/ob* mice. The difference at 2 months of age was 13% (*p* < 0.05, two way-ANOVA, Sidak’s multiple comparison test), while at 4 and 6 months, A2-GPE was 34% and 37% lower, respectively (*p* < 0.01) (Fig. 4*C*). The difference at age 8 months was not statistically significant (*p* > 0.05). Since bisretinoids are the source of SW-AF that is imaged *in vivo*, we used the qAF approach to quantify SW-AF (qAF) in black-coated *ob/ob* mice (15, 55). As expected, we observed an age-related increase in qAF in the *ob/ob* mice (Fig. 4*D*), but qAF intensities were not different in the *ob/ob* mice as compared to C57BL/6J mice at 2 and 4 months of age nor was there a difference in near-infrared fundus autofluorescence (Fig. 4*E*) that for the most part originates from RPE melanin (56). Interestingly, measurements of ONL thicknesses revealed thinning in the *ob/ob* mice relative to C57BL/6J mice at 2 and 4 months of age (Fig. 4, *F*–*H*). Whether photoreceptor cell degeneration obfuscated a tendency toward elevated bisretinoid has not been determined. And finally, in a fluorescence assay of PE, levels were not significantly different in *ob/ob* mice *versus* C57BL/6J mice at age 3 months (two-way ANOVA, Sidak’s multiple comparison test) (Fig. 3*B*).

## Discussion

Numerous publications have reported on the effects on retina of an impaired retinoid cycle (57). By comparison, less is known regarding the effects of increased circulating vitamin A on the visual cycle. The salient finding of this study in mice is that an HFD not only leads to elevated Rbp4, retinol, and glucose in plasma, it also contributes to elevated levels of bisretinoids in retina. Here, the latter increase was measured both by fundus imaging and by quantitative HPLC/UPLC.

For transport in the circulatory system, retinol forms a holo-enzyme complex with RBP4 and TTR (45, 46). Approximately 85% of serum RBP4 is found within this complex with the remaining being apo-RBP4 (58). At target cells, RBP4-TTR binds to cell surface STRA6 (stimulated by retinoic acid 6) (59), and retinol enters the RPE cell where it binds to cellular retinol binding protein-1. It is also now evident that calcium/calmodulin regulates vitamin A transport activity by STRA6 (60). Specifically, calcium/calmodulin suppresses STRA6-mediated vitamin A uptake by promoting the binding of apo-RBP to STRA6. Binding of apo-RBP to STRA6 not only thwarts the binding of holo-RBP to STRA6, a process that is required for vitamin A uptake, binding also promotes vitamin A efflux through STRA6. Within the RPE, *at*RE is converted to the visual chromophore 11-*cis*-retinal (61). There is evidence that RBP4 also serves as a transporter of fatty acid (62).

Efficient absorption of fat-soluble vitamin A in the intestine depends on the fat-solubilizing properties of bile acids. Specifically, within the lumen of the small intestine, bile acids form mixed micelles with phospholipids that promote solubilization and absorption of dietary lipids; by incorporating vitamin A in these particles, absorption of the latter is also facilitated (63). Hence, dietary retinol absorbed by intestinal enterocytes can be converted to retinyl esters and incorporated into chylomicron particles (64, 65). The latter lipoprotein particles are composed of a central lipid core of triglycerides and as with other lipoproteins, chylomicrons carry esterified cholesterol and phospholipids. These chylomicrons serve to transport vitamin A in addition to its transport as retinol bound to RBP4. Under conditions of high fat intake, increases in liver fatty acid–binding protein L-FABP and microsomal triglyceride transfer protein, both of which are responsible for the transport of fatty acids from the cytosol to the endoplasmic reticulum and incorporation of lipid into pre-chylomicrons, may directly influence the quantity of chylomicrons reaching the circulatory system (66). When remodeling serum chylomicrons, lipoprotein lipase acts not only on lipid, it also hydrolyzes retinyl esters; the product of this lipolysis is retinol which is then taken up by extrahepatic cells (58). This alternative system of vitamin A transport may explain why in *Rbp4* null mice, normal visual functioning is eventually observed (∼ age 5 months) if vitamin A intake is sufficient (65). In *STRA6*^*−/−*^ mice, ocular retinoid concentrations are low in the young mouse but by 8 months of age reach levels that are 50% of the content in WT mice (67, 68).

The phenotype of mice deficient in leptin (*ob/ob)* differed from that of the HFD-fed mice. While both HFD-fed mice and *ob/ob* mice exhibited elevated serum Rbp4, the *ob/ob* mice acquired the weight gain in the absence of high fat intake and did not exhibit increased bisretinoid accumulation. This difference suggests that the HFD *per se* rather than the weight gain explains the bisretinoid increase. In some studies but not others, obesity in human subjects has also been associated with increased serum RBP4 (58).

The development of obesity in humans is multifactorial; it depends on genetic susceptibility, increased caloric intake, and inadequate energy expenditure. Conversely, body weight gain due to leptin deficiency (*ob/ob*) happens as a result of enhanced food intake even on a normal diet. Thus, the HFD-induced obesity is more representative of the human condition.

Diet-induced obesity has been reported to have other effects on retina. For instance, in C57BL/6J mice, an HFD is associated with reductions in ERG amplitudes (69, 70), and in the setting of disrupted cholesterol metabolism, an HFD accelerated rod degeneration (71). Basal laminar sub-RPE deposits form in C57BL/6J WT and APO B100 transgenic mice placed on an HFD for several months (72, 73, 74, 75). Mice deficient in apolipoprotein E4 when placed on a high fat cholesterol-enriched diet exhibit abnormal sub-RPE Aß-containing deposits (76) as do mice deficient in peroxisome proliferator-activated receptor-g coactivator 1-alpha when fed a diet high in fat (55). In mice lacking nuclear factor erythroid 2-related factor 2, an HFD caused accelerated RPE atrophy (77). One limitation of the current study was that all HFD-fed mice were male, this approach being customary (78, 79, 80).

One may have expected that the increase in bisretinoid fluorophores would reflect an increase in ocular retinoids. Nevertheless, while plasma retinol was elevated and bisretinoid formation was increased, we did not detect a change in ocular retinoids (all-*trans*-retinol, all-*trans*-retinyl ester, all-*trans*-retinal, 11-*cis*-retinal). On one hand, this finding is consistent with the work of others revealing that obesity in mice and humans is associated with unchanged tissue levels of vitamin A (47) even when the diets contain adequate amounts of retinol or retinyl palmitate (78). On the other hand, however, the magnitude of bisretinoid accumulation is known in many cases to mirror retinoid cycle kinetics (29, 31). Moreover, we have observed that a disparity in ocular retinoid, for instance due to the Rpe65-Leu450Met variant, can manifest as a more pronounced difference in bisretinoid accumulation (81) due to a cumulative effect. Thus, measurements of bisretinoid products of the visual cycle serve as an index of visual cycle activity over the long term even when changes in retinoid levels may be difficult to detect by measuring individual retinoids at any given time (82, 83).

The formation of *N*-retinylidene-PE by a Schiff base linkage between retinaldehyde and PE (1:1 ratio) in the photoreceptor outer segment membrane is the first step in the nonenzymatic process by which bisretinoids form. *N*-retinylidene-PE is also the ligand recognized by ATP-binding cassette transporter A4, a member of the ABC family of lipid transporters, that serves to deliver retinaldehyde to cytosolic retinol dehydrogenases which reduce retinaldehyde to the nonreactive retinol form (84, 85, 86, 87, 88). In previous work, we carried out a study in which we varied the ratio of all-*trans*-retinaldehyde to PE in synthetic mixtures to determine how these two precursors might affect the formation of A2PE, an intermediate in the A2E biosynthetic pathway. We found that the yield of A2PE increased when the ratio of equivalents of all-*trans*-retinal to PE was increased from 1:2 to 4:2 with no further increase being observed at a ratio of 8:2 (1). Conversely, increasing the concentration of PE from an equivalents ratio of 4:1 through 4:8 (all-*trans*-retinal to PE) resulted in a steady increase in A2PE synthesis. This finding indicated that the concentration of PE has a pronounced effect on the rate of A2PE synthesis. An increase in PE in the reaction mixture also favored the formation of all-*trans*-retinal dimer (1). These findings are consistent with a relationship between PE content and bisretinoid formation observed in association with an HFD in the current work.

In summary, we show that lipid metabolism intersects with the visual cycle and bisretinoid lipofuscin formation. We propose that dysregulation of vitamin A aldehyde in association with an HFD can lead to increased formation of bisretinoid fluorophores that constitute the lipofuscin of retina. An alternative or additional mechanism could involve HFD-associated changes in the PE content of photoreceptor cells. In addition to the current findings, it is reported that mice reared on an HFD exhibit changes in the relative proportion of omega-6 and omega-3 polyunsaturated fatty acid precursors (linoleic and alpha-linolenic acids) incorporated in the retina (89). Genes involved in lipid metabolism are also amongst 34 loci implicated in age-related macular degeneration susceptibility (90). The relationship between retinal disease and lipid metabolism is important: 70% of American adults are overweight (91) and obesity is a major contributor to disease burden. This investigation adds to existing notions regarding rates of bisretinoid formation.

## Experimental procedures

### Animals and treatments

Diet-induced obese mice (black C57BL/6J; Stock# 380 and albino C57BL/6J^c2j^) were obtained from The Jackson Laboratory (JAX) at ages 4 to 12 months. Black male C57BL/6 mice and albino male C57BL/6J^c2j^ were fed an HFD with 60% of kcal from fat (4.0 IU vitamin A acetate/gm chow). Male black C57BL/6 mice also received standard diet of 10% kcal from fat (4.0 IU vitamin A acetate/gm chow), and albino C57BL/6J^c2j^ were fed a standard diet of 16.6% kcal from fat (20 IU vitamin A acetate/gm chow). Leptin-deficient *ob/ob* mice (*B6.V-Lep/Job/ob*) were obtained from JAX. Ob/ob mice and littermate controls were fed a diet with 13.1% kcal from fat (15 IU vitamin A acetate/gm chow). Eyes from 36-month-old DIO mice were a gift from Drs J. L. Walewski and P. D. Berk, Department Medicine, Columbia University Medical Center. C57BL/6J and C57BL/6J^c2j^ mice carry the methionine variant at amino acid 450 (Rpe65-Met450). WT mice, black C57BL/6J, or albino C57BL/6J^c2j^ mice were obtained from JAX. For consistency with published work, mice employed for studies of the effects of an HFD were male. Estrogen-associated effects on food intake and energy expenditure in female mice leads to more variable data (92). Blood glucose was measured on the day of shipment and body weight was determined immediately upon arrival.

Animal protocols were approved by the Institutional Animal Care and Use Committees of Columbia University and all procedures complied with the Association for Research in Vision and Ophthalmology Statement for the Use of Animals in Ophthalmic and Vision Research. All mice had free access to water, were fed *ad libitum* throughout, and were maintained in a temperature-controled room at 21 to 23 °C under cyclic light (12 h: 12 h light-dark cycle).

### Quantitative HPLC and UPLC of bisretinoids

Mouse eyecups were homogenized and extracted with either A or B (A: homogenized in dulbecco’s phosphate buffered saline (DPBS) and extracted with chloroform/methanol, 1:1; B: homogenized and extracted in chloroform/methanol, 1:1) using a tissue grinder. Solvent (200 μl) was added to each eyecup. To exceed the lower limit of quantification for each bisretinoid, eyes were pooled according to mouse strain (pigmentation, age, and genotypes), and samples were extracted according to procedure A or B above. Samples were extracted five times and then centrifuged at 4000*g* for 3 min. The extract was concentrated and redissolved in 50% methanolic chloroform. Molecular quantities of bisretinoids were presented as pmoles per eye. Bisretinoids were analyzed and quantified by reversed-phase HPLC (A2E, iso-A2E, A2-DHP-PE, atRALdi-PE) (4–10 eyes/sample as indicated) using an Alliance System (Waters Corp) and Atlantis dC18 column. Gradients of water and acetonitrile with 0.1% of TFA were used for mobile phase; 75 to 90% acetonitrile (0–30 min); 90 to 100% acetonitrile (30–40 min); 100% acetonitrile (40–80 min) with a flow rate of 0.5 ml/min. The bisretinoid A2-GPE was analyzed using a Waters Acquity UPLC-MS system and Acquity BEH phenyl column (1–4 eyes/sample as indicated) with a mobile phase of acetonitrile/water (1:1) and isopropanol/acetonitrile (9:1), both with 0.1% formic acid (UPLC condition a: 0–50 min, 100–55% acetonitrile/water in isopropanol/acetonitrile; 50–110 min, 55–35% acetonitrile/water in isopropanol/acetonitrile; flow rate of 0.2 ml/min) (23). Molar quantities per eye were calculated by comparison to synthesized standards. The pyridinium bisretinoid A2E and its *cis* isomer, iso-A2E were measured separately and summed (A2E).

### Quantitative UPLC analysis of ocular and plasma retinoids

For extraction of ocular retinoids, frozen mouse eyecups (1 eye/sample) were homogenized and derivatized with *O*-ethylhydroxylamine (100 mM) in DPBS (, pH 6.5, without CaCl_2_ and MgCl_2_) (method 1) or with *O*-ethylhydroxylamine (100 mM) and acetonitrile (method 2) on ice under dim red light. After vortexing, samples were allowed to stand for 15 min at 4 °C. Methanol (1 ml) was added (method 1) or was not added (method 2), and the ocular retinoids were extracted with hexane (3 ml, 3 times) and then resuspended in 20 μl of acetonitrile for UPLC analysis (93). Mouse plasma samples collected using EDTA were centrifuged at 1000*g* for 10 min at 4 °C. For extraction of retinoids, 1 ml of methanol and 120 μl of 1M *O*-ethylhydroxylamine in DPBS (pH 6.5, without CaCl_2_ and MgCl_2_) were added to 200 μl of plasma (final concentration of *O*-ethylhydroxylamine, 100 mM). Plasma retinoids were extracted with hexane (5 ml, 2 times) and then resuspended in 10 μl of acetonitrile for UPLC analysis. For quantitative retinoids analysis, a Waters Acquity UPLC-PDA system was used with a CSH C18 column (1.7 μm, 2.1 × 100 mm) and gradients of water (A) and acetonitrile (B) with 0.1% of formic acid as described (32). Molar quantities per eye were calculated based on standard curves determined spectrophotometrically. Peak areas were calculated using Waters Empower Software (https://www.waters.com/nextgen/us/en/products/informatics-and-software/waters_connect.html), and results were analyzed in Excel (Microsoft). *O*-Ethylhydroxylamine was used to convert retinaldehyde into a stable oxime product for accurate quantitation.

### Measurements of RBP4 protein by ELISA

Mouse plasma samples collected using EDTA were centrifuged at 1000*g* for 10 min at 4 °C. The plasma samples were diluted with the sample diluent buffer provided in the kit in a ratio of 1 either to 500,000 or 1,000,000 and measured using a commercial RBP4 mouse ELISA kit (Abcam) following the manufacturer’s guidelines. Absorbance was read at 450 nm, and corresponding RBP4 protein level (microG/ml) was determined by reference to a standard curve (absorbance as a function of log concentration) and the use of a four-parameter fit algorithm (94). A control blank value was subtracted from all readings.

### Fundus imaging

Mice were anesthetized, pupils were dilated, the cornea was lubricated, and mice were positioned as previously described (15). Fundus AF images (55° wide field lens; 0.98-mm detection pupil) at 488 nm and 790 nm excitation were obtained with a confocal scanning laser ophthalmoscope (Spectralis HRA; Heidelberg Engineering) with laser power set at approximately 280 μW and sensitivity at 100 and 105, respectively, after visual pigment was bleached for 20 s. Nine successive frames were acquired at 488 nm excitation with the high-speed mode, and frames were saved in non-normalized mode. A mean of 100 frames was obtained at 790-nm excitation with high-resolution automatic real-time mode and resized with Photoshop CS4 (Adobe Systems, Inc) to 768 × 768 pixels, the same as high-speed mode images. Near-infrared reflectance images (820 nm) were also acquired.

### Quantitative fundus autofluorescence

Using a dedicated image analysis program written in IGOR (Wavemetrics), mean gray levels (GLs) were calculated from eight predefined segments around the optic disc, and blood vessels were excluded by histogram analysis. qAF at 488-nm excitation was calculated by normalization to the GL of the reference after subtraction of zero light (GL_0_) and inclusion of a reference calibration factor (15). Fluorescence intensities at 790 nm were calculated by subtracting the minimal GL of optic nerve head (ONH) measured by ImageJ software (http://imagej.nih.gov/ij/; provided in the public domain by the National Institutes of Health).

### Measurement of ONL thickness

Following sacrifice and enucleation, eyes were immersed in 4% paraformaldehyde for 24 h at 4 °C. Sagittal paraffin serial sections of mouse retina were prepared and stained with H&E. The section most centrally positioned in the ONH was selected and imaged with the 20× objective using a digital imaging system (Leica Microsystems; Leica Application suite), and composite images were created in Photoshop CS5. ONL thickness was then measured at 200 micron intervals superior and inferior to the edge of the ONH along the vertical meridian (95). The measurements were made using the Photoshop CS5 ruler tool and a custom measurement scale. ONL width in pixels was converted to microns (1 pixel: 0.23 μm). For groups of mice at defined ages, mean ONL thickness at each position along the vertical meridian was plotted as a function of eccentricity from the ONH. ONL area was calculated as the measurement interval of 0.2 mm multiplied by the sum of ONL thicknesses in superior and inferior hemiretina.

### Assay of PE

Dissected ocular tissues were homogenized in a 5% (v/v) solution of peroxide free Triton X-100 in water using an ultrasonic homogenizer. The homogenate solution was heated to 80 °C for 5 to 10 min and then cooled down to room temperature. The latter process was repeated to solubilize all lipids. After centrifugation (10,000*g*, 10 min, 4 °C), samples were collected from supernatant. PE content was measured in the samples using a fluorescence assay kit whereby a PE converter hydrolyzes PE to an intermediate which converts a colorless probe to a fluorescent product *via* enzymatic reaction (Ex/Em: 535/587) (ab241005 PE assay kit, Abcam).

### Statistical analysis

Statistical analysis was carried out using GraphPad Prism 8.0, and *p* < 0.05 was considered significant. Effect size (d) was calculated as (mean1−mean2)/pooled SD (96).

## Data availability

All of the data are in the manuscript. Histological images and chromatograms are available on request.

## Conflicts of interest

The authors declare that there are no conflicts of interest related to the contents of this article.
